# Genomic Prediction of Genotypic Effects with Epistasis and Environment Interactions for Yield-Related Traits of Rapeseed (*Brassica napus* L.)

**DOI:** 10.3389/fgene.2017.00015

**Published:** 2017-02-21

**Authors:** Xiang Luo, Yi Ding, Linzhong Zhang, Yao Yue, John H. Snyder, Chaozhi Ma, Jun Zhu

**Affiliations:** ^1^National Key Laboratory of Crop Genetic Improvement, National Center of Rapeseed Improvement in Wuhan, Huazhong Agricultural UniversityWuhan, China; ^2^Institute of Bioinformatics, Zhejiang UniversityHangzhou, China; ^3^Economic and Technical College, Anhui Agricultural UniversityHefei, China

**Keywords:** genomic prediction, genotypic effects, epistasis, agronomic traits, *B. napus*

## Abstract

Oilseed rape (*Brassica napus*) is an economically important oil crop, yet the genetic architecture of its complex traits remain largely unknown. Here, genome-wide association study was conducted for eight yield-related traits to dissect the genetic architecture of additive, dominance, epistasis, and their environment interaction. Additionally, the optimal genotype combination and the breeding value of superior line, superior hybrid and existing best line in mapping population were predicted for each trait in two environments based on the predicted genotypic effects. As a result, 17 quantitative trait SNPs (QTSs) were identified significantly for target traits with total heritability varied from 58.47 to 87.98%, most of which were contributed by dominance, epistasis, and environment-specific effects. The results indicated that non-additive effects were large contributions to heritability and epistasis, and also noted that environment interactions were important variants for oilseed breeding. Our study facilitates the understanding of genetic basis of rapeseed yield trait, helps to accelerate rapeseed breading, and also offers a roadmap for precision plant breeding via marker-assisted selection.

## Introduction

Rapeseed (*Brassica napus* L.; AACC, 2*n* = 38) is one of the most important sources of vegetable oil in the world. Owing largely to the development of high yielding varieties of rapeseed in breeding programs, now hybrid rape has the largest market share due to its superior performance for multiple agronomic traits (Liu et al., 2002). While the genetic architectures of complex traits is still vague.

Genome-wide association studies (GWAS) are now becoming one of the standard methodology for revealing the genetic variation of complex traits for human, plants, and animals (Clark, 2010; Cockram et al., 2010; Zhang et al., 2010; Flint and Eskin, 2012; Segura et al., 2012; Li et al., 2013). Several GWAS studies in *B. napus* have been reported for seed quality traits (Li F. et al., 2014; Liu S. et al., 2016), branch angle (Liu J. et al., 2016), flower time (Wang et al., 2016; Xu et al., 2016), and yield-related traits mainly containing seed weight (Li F. et al., 2014), plant height (Li et al., 2016), primary branch number (Li et al., 2016) in recent years. However, these potential loci identified with GWAS methods only explain a small amount of phenotypic variance (4.3~25.17% for seed quality traits, 16.47~20.51% for branch angle, 5.62~15.75% for flower time and 3.72~13.87% for yield-related traits). A probable reason for the low heritability detected with GWAS is that only additive models are applied but not considering dominance, epistasis (Zuk et al., 2012; Hemani et al., 2013). In a study of *Drosophila melanogaster* populations, about 50% of phenotypic variation in adult olfactory behavior was assigned to genotype-by-environment (G×E) interaction (Sambandan et al., 2008). Similarly, G×E or epistatic interactions could explain considerable proportion of variance of flowering traits in rice (Uwatoko et al., 2008) and in *Arabidopsis* (Caicedo et al., 2004; El-Lithy et al., 2006). Thus, trying to find the so-called “missing heritability” could help to efficiently dissect the genetic mechanism of complex traits (Manolio et al., 2009; Ingvarsson and Street, 2011; Resende et al., 2012).

In the earlier studies of QTL mapping, epistasis was observed for the resistance to *Sclerotinia sclerotiorum* in rapeseed, and the additive by additive interactions were the predominant type of epistasis (Zhao and Meng, 2003). Additionally, total 190 plant architecture (PA)-related candidate genes for 91 unique PA QTLs and 2,350 plant yield (PY) epistatic interaction loci-pairs were identified, which explain 2.8–51.8 and 5.2–23.6% of phenotypic variation, respectively (Cai et al., 2016). However, the published studies relied on kinds of low density traditional markers. Recently, the revolution of inexpensive sequencing has been used for the identification of high-throughput genomic SNPs (Ganal et al., 2009), and a new mixed linear model approach has been developed to dissect the genetic architecture of multiple loci by partitioning into additive, dominance, epistasis, and environmental interactions (Zhang et al., 2015).

Rapeseed seed yield is usually determined by several factors including plant height (PH), main inflorescence length (IL), branch number (BN), number of seeds per silique (SS), effective silique number on main inflorescence (ISN), thousand seed weight (TSW), biomass yield per plant (BY), and seed yield per plant (SY). In the present study, the eight yield-related traits of 367 experimental materials were detected in two environments. GWAS performed with high-throughout SNPs was firstly used to find SNPs with additive, dominance, epistasis and environmental interactions for these traits. Additionally, prediction of genetic effects was conducted based on the detected quantitative trait SNPs (QTSs) of eight agronomic traits to predict high-yield genotype combination. The study will provide a better understanding of the genetic mechanism of yield-related traits, and the QTSs are beneficial for efficiently selecting ideal parental lines for breeding new hybrid rapeseed.

## Materials and methods

### Plant materials and field experiments

To generate genetic population used for the association studies, we adopted Yu5 (L155) and Zheyou18 (L157) as female parents to pollinate with another 149 male parents (L1-L149) and finally harvested 216 F_1_ hybrids including 97 hybrids taken L155 as female and 119 hybrids taken L157 as female. All the lines came from China and adapted to the middle reaches of the Yangtze River agro-climatic conditions, China. The 367 experimental materials, including 151 inbred lines and 216 F_1_ hybrids were grown in farm fields in Wuhan (29.58° N, 113.53° E) and Xiangyang (32.01° N, 112.08° E) in Hubei province, China, under normal conditions for crop production during the rapeseed growing season of 2012–2013. The annual mean temperature of Wuhan was 1~2° higher than Xiangyang, and the average rain days of Wuhan was 3 days more than Xiangyang during the rapeseed growing season of 2012–2013.

In 2012, all trials were designed as randomized complete blocks with three replications in each environment. Each plot consisted of three rows of 3.5 m length with 0.25 m distance between rows. During the last 5 days of September and the first 5 days of October, seeds were sown with a distance of 0.15 cm between plants in each row. The management of the field experiments was performed in accordance with local standard production practices. At maturity, 12 plants in the middle row were harvested (randomly within this row) from each plot for evaluation of the following eight quantitative traits: plant height (PH), main inflorescence length (IL), branch number (BN), number of seeds per silique (SS), effective silique number on the main inflorescence (ISN), thousand seed weight (TSW), biomass yield per plant (BY), and seed yield per plant (SY). Measurements for PH, IL, BN, SS, ISN, TSW, BY, and SY were completed as described by Shi et al. (2011).

### Genotyping and quality control

Genotyping of the entire inbred line population was performed commercially by Emei Tongde Co. (Beijing) using the Brassica 60 K Illumina® Infinium SNP array (http://www.illumina.com/technology/infinium_hd_assay.ilmn) according to the manufacturer's protocol. The SNP data were clustered and called automatically using Illumina BeadStudio genotyping software. SNPs with QQ or qq frequencies equal to zero, call frequency <0.9, or minor frequency <0.05 were excluded. The remaining SNPs were scrutinized visually, and those SNPs not showing three clearly defined clusters representing the three possible genotypes (QQ, Qq, qq) were also excluded. A total of 33,689 SNP markers among the 367 lines and hybrids that met the QC criteria were used for the association analyses.

The phenotypes of 8 traits were filtered based on the following two criteria: (1) the frequency of missing data for each subject <0.1; (2) outliers were discarded according to a distribution-based outlier detection of residuals (|ε − μ_ε_|/ σ_ε_ > 3) by QQ plot (Quantile-Quantile plot) for each trait (Figure S1).

### Statistical analysis

The genetic model for the phenotypic value of the *k*-th line or hybrid in the *h*-th environment (*y*_*hk*_) can be expressed by the following linear mixed model:

(1) $$\begin{array}{l} y_{hk}=\mu +\sum_{i}a_{i}x_{A_{ik}}+\sum_{i}d_{i}x_{D_{ik}}+\sum_{i\;<\;j}aa_{ij}x_{AA_{ijk}}\\ \;+\sum_{i\;<\;j}ad_{ij}x_{AD_{ijk}}+\sum_{i\;<\;j}da_{ij}x_{DA_{ijk}}+\sum_{i\;<\;j}dd_{ij}x_{DD_{ijk}}+e_{h}\\ \;+\sum_{i}ae_{ih}u_{AE_{ihk}}+\sum_{i}de_{ih}u_{DE_{ihk}}+\sum_{i\;<\;j}aae_{ijh}u_{AAE_{ijhk}}\\ \;+\sum_{i\;<\;j}ade_{ijh}u_{ADE_{ijhk}}+\sum_{i\;<\;j}dae_{ijh}u_{DAE_{ijhk}}\\ \;+\sum_{i\;<\;j}dde_{ijh}u_{DDE_{ijhk}}+\varepsilon_{hk} \end{array}$$

where μ is the population mean; ***a***_*i*_ is the additive effect of the *i*-th locus with coefficient ***u***_*A*_*ik*__ (1 for homozygote major alleles *QQ* and −1 for homozygote minor alleles *qq*); ***d***_*i*_ is the dominance effect of the *i*-th locus with coefficient ***u***_*D*_*ik*__ (1 for heterozygote *Qq*, 0 for homozygotes *QQ* and *qq*); ***aa***_*ij*_, ***ad***_*ij*_, ***da***_*ij*_, and ***dd***_*ij*_ are the digenic epistasis effects with coefficients of random variables ***u***_*AA*_*ijk*__ (1 for *QQ* × *QQ* and *qq* × *qq*, −1 for *QQ* × *qq* and *qq* × *QQ*), ***u***_*AD*_*ijk*__ (1 for *QQ* × *Qq*, −1 for *qq* × *Qq*), ***u***_*DA*_*ijk*__ (1 for *Qq* × *QQ*, −1 for *Qq* × *qq*), and ***u***_*DD*_*ijk*__ (1 for *Qq* × *Qq*); ***e***_*h*_ is the random effect of the *h*-th environment; ***ae***_*ih*_ is the additive × environment interaction effect of the *i*-th locus in the *h*-th environment with coefficient ***u***_*AE*_*ihk*__; ***de***_*ih*_ is the dominance × environment interaction effect of the *i*-th locus in the *h*-th environment with coefficient ***u***_*DE*_*ihk*__; ***aae***_*ijh*_, ***ade***_*ijh*_, ***dae***_*ijh*_, and ***dde***_*ijh*_ are the digenic epistasis × race interaction effects in the *h*-th ethnic population with coefficients of random variables (***u***_*AAE*_*ijhk*__, ***u***_*ADE*_*ijhk*__, ***u***_*DAE*_*ijhk*__, and ***u***_*DDE*_*ijhk*__); and ε_*hk*_ is the residual effect of the *k*-th line or hybrid in the *h*-th environment.

Heritability of individual genetic effects were estimated by $h_{g}^{2}=\alpha \sigma_{g}^{2}/V_{P}$ (α = 2 for additive effect, α = 1 for dominant effect, α = 4 for additive × additive, α = 2 for additive × dominant or dominant × additive, α = 1 for dominant × dominant), where phenotypic variance (*V*_*P*_) is the sum of genetic variance (*V*_*G*_), genetic by environment interaction variance (*V*_*GE*_), and residual variance (*V*_ε_),

(2) $$\begin{array}{l} V_{P}=V_{G}+V_{GE}+V_{\varepsilon }\\ \;=(V_{A}+V_{D}+V_{I})+(V_{AE}+V_{DE}+V_{IE})+V_{\varepsilon }\\ \;=(V_{A}+V_{D}+V_{AA}+V_{AD}+V_{DA}+V_{DD})+\\ \;(V_{AE}+V_{DE}+V_{AAE}+V_{ADE}+V_{DAE}+V_{DDE})+V_{\varepsilon } \end{array}$$

The total heritability was estimated by

$$\begin{array}{l} h_{G\;+\;GE}^{2}=(h_{A}^{2}+h_{D}^{2}+h_{I}^{2})+(h_{AE}^{2}+h_{DE}^{2}+h_{IE}^{2})\\ \;=(h_{A}^{2}+h_{D}^{2}+h_{AA}^{2}+h_{AD}^{2}+h_{DA}^{2}+h_{DD}^{2})\\ \;+(h_{AE}^{2}+h_{DE}^{2}+h_{AAE}^{2}+h_{ADE}^{2}+h_{DAE}^{2}+h_{DDE}^{2})\\ \;=\sum_{i}h_{a}^{2}+\sum_{i}h_{d}^{2}+\sum_{i\;<\;j}h_{aa}^{2}+\sum_{i\;<\;j}h_{ad}^{2}+\sum_{i\;<\;j}h_{da}^{2}\\ \;+\sum_{i\;<\;j}h_{dd}^{2}+\sum_{i}h_{ae}^{2}+\sum_{i}h_{de}^{2}+\sum_{i\;<\;j}h_{aae}^{2}+\sum_{i\;<\;j}h_{ade}^{2}\\ \;+\sum_{i\;<\;j}h_{dae}^{2}+\sum_{i\;<\;j}h_{dde}^{2} \end{array}$$

We used the GMDR module (Generalized Multifactor Dimensionality Reduction; Qi et al., 2013) in the *QTXNetwork* software (http://ibi.zju.edu.cn/software/QTXNetwork/) to scan 33,689 SNP markers in 367 subjects for 1D~3D significant candidate SNP markers, and obtained 539 candidate SNPs (260 in the A genome, 262 in the C genome, and 17 in the Scaffold group). The QTS mapping module in the *QTXNetwork* was then used to dissect the genetic architecture of the eight agronomic traits of oilseed rape (Zhang et al., 2015). Significant SNPs associated with phenotypic variants were analyzed by setting a total of 2,000 permutation tests to calculate the critical *P*-value for controlling the experiment-wise type I error. The effects were predicted by using a Markov Chain Monte Carlo method with 20,000 Gibbs sampler iterations (et al., 2007). The correlation coefficient (***R***_Ŷ_) between predicted breeding values and phenotypic values was estimated for each trait. Based on the predicted genetics effects of SNP loci for eight traits, we predicted total genotypic effects for the best lines and the best hybrids of the mapping population, and also predicted genotypic effects of superior lines and superior hybrids to inform further selection decisions (Yang and Zhu, 2005).

## Results

### Estimated heritability and predicted genetic effects

**Figure 1:**
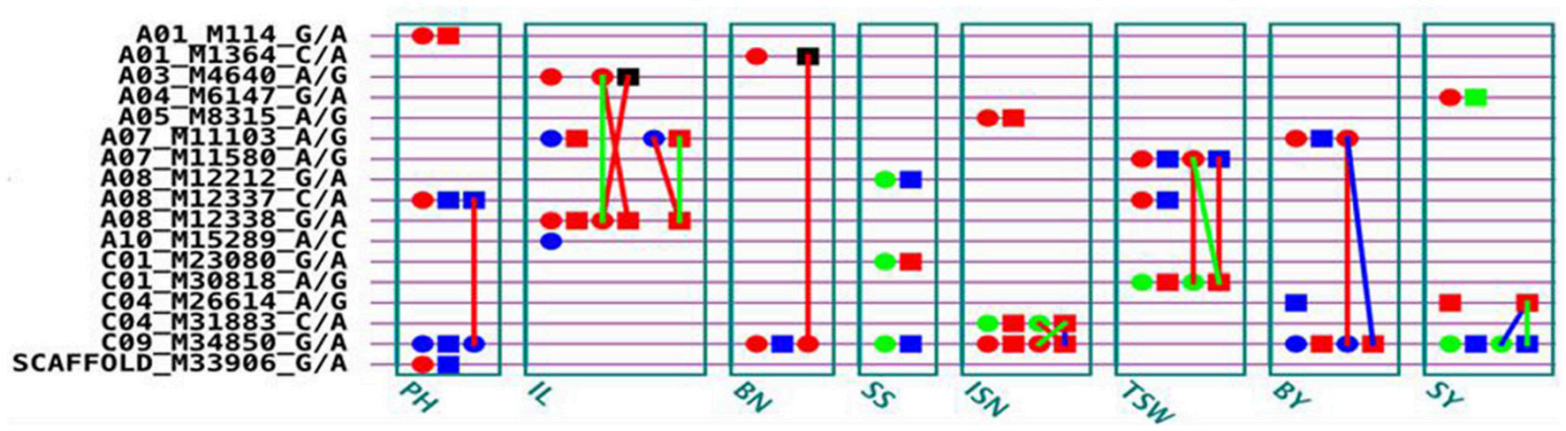
**G×G plot of detected significant QTSs (***P***_**EW**_ < 0.05) for eight traits**. Circle, QTS with additive effect; Square, QTS with dominant effect; Line between two QTSs, epistasis effect; Red color, QTS with general effects for two environments; Green color, QTS with environment-specific effects; Blue color, QTS with both general and environment-specific effects; Black color, QTS with significant epistasis effects but without detected individual effects.

We conducted a genome wide association study for eight yield-related traits of *B. napus* on a population including 151 inbred lines and 216 F_1_ hybrids obtained from mating two female parents (L155 and L157) to other 149 inbred lines as male parents. Eight yield-related traits were investigated in our study by using a full genetic model with genetic effects of additive, dominance, epistasis, and their environment interactions. A total of 17 QTSs controlling eight yield traits were detected: 4 QTSs for PH, 2 QTSs for BN, 3 QTSs for IL, SS, ISN, TSW, BY, and SY, respectively (Figure 1 and Table S1). Some loci exhibited pleiotropic effects, including C09_M34850_G/A for six traits (BN, BY, ISN, PH, SS, and SY), A07_M11103_A/G for both BY and IL, A08_M12337_C/A for both PH and TSW, and C04_M26614_A/G for both BY and SY.

Estimated heritability and correlation coefficients ($R_{\hat{Y}}$) between total genotypic values of detected QTSs and phenotypic values for the eight traits are listed in Table 1. The total heritability ranged from 58.47 to 87.98%, and was contributed by various types of genetic variance effects. With the exception of three yield traits (SS, BY, and SY), which were sensitive to the environment ($h_{GE}^{2}\overset{\wedge }{=}$ 60.24% for SY, 59.75% for BY, and 49.57% for SS), the other five traits were quite stable across the two environments ($h_{GE}^{2}\overset{\wedge }{=}$ 4.14~27.40%). With the exception of the SS trait, epistasis effects contributed a large portion of total heritability ($h_{I+IE}^{2}\overset{\wedge }{=}$ 26.07~62.14%). The correlation coefficient of genetic prediction with phenotype ($R_{\hat{Y}}$ = 0.501~0.899) for each trait was very close to the estimated heritability ($h_{T}^{2}\overset{\wedge }{=}$ 58.47~87.98%), indicating that this statistic approach would be quite efficient for predicting the best lines/hybrids, and selecting the superior lines/hybrids by using the predicted genetic effects.

**Table 1:** **Estimates of heritability and correlation coefficient of detected QTSs for eight traits**.

**Trait**	***$h_{A}^{2}$(%)***	***$h_{D}^{2}$(%)***	***$h_{I}^{2}$(%)***	***$h_{AE}^{2}$(%)***	***$h_{DE}^{2}$(%)***	***$h_{IE}^{2}$(%)***	***$h_{T}^{2}$(%)***	***R_Ŷ_***
PH	12.04	13.33	50.30	2.57	6.82	0.00	85.06	0.82
IL	4.15	20.75	53.01	3.92	0.00	6.15	87.98	0.73
BN	3.87	25.94	44.33	0.00	4.14	0.00	78.28	0.82
SS	0.00	8.90	0.00	35.91	13.66	0.00	58.47	0.50
ISN	3.86	35.52	5.79	3.03	0.00	24.37	72.57	0.74
TSW	17.23	8.32	15.11	2.15	7.57	10.96	61.34	0.62
BY	4.68	5.00	14.51	2.99	28.88	27.88	83.94	0.89
SY	1.27	11.11	13.96	5.51	6.55	48.18	86.58	0.90

*$h_{A}^{2}$, heritability of additive effects; $h_{D}^{2}$, heritability of dominance effects; $h_{I}^{2}$, heritability of epistasis effects (AA, AD, DA, and DD); $h_{AE}^{2}$, heritability of environment- specific additive effects; $h_{DE}^{2}$, heritability of environment-specific dominance effects; $h_{IE}^{2}$, heritability of environment-specific epistasis effects (AAE, ADE, DAE, and DDE); $h_{T}^{2}$, total heritability of all genetic effects. R_Ŷ_, correlation coefficient between phenotypic values and predicted genotypic values*.

*PH, plant height; IL, main inflorescence length; BN, branch number; SS, number of seeds per silique; ISN, effective silique number on main inflorescence; TSW, thousand seed weight; BY, biomass yield per plant; SY, seed yield per plant*.

Highly significant (experiment-wise *P*_*EW*_–value < 10^−5^) predicted genetic effects of 16 QTSs are presented in Table 2. Among the eight traits studied, plant height (PH) had the highest heritability ($h_{T}^{2}\overset{\wedge }{=}$ 85.06%) mainly contributed by epistasis ($h_{I}^{2}\overset{\wedge }{=}$ 50.30%). There was one locus (G/A of C09_M34850) detected with large and positive dominance effects. Homozygotes of this locus (G/G) could also increase plant height. The additive and dominance effects ($a\overset{\wedge }{=}$ −7.107 of G/G, $d\overset{\wedge }{=}$ −3.565 of G/A) were negative for locus Scaffold_M33906_G/A, which could be used in selecting for decreased plant height. Heterozygote C/A of A08_M12337 and homozygote G/G of C09_M34850 had a negative dominance × additive epistasis effect ($da\overset{\wedge }{=}$ −11.003), which could dramatically decrease plant height; however this large epistasis effect was counteracted by their main effects. Instead, the combination of homozygotes for major-allele C/C of A08_M12337 and minor-allele A/A of C09_M34850 implicated a lower plant height (Figure S2A). Epistasis effects were also the most important genetic effects on main inflorescence length (IL) ($h_{I}^{2}\overset{\wedge }{=}$ 53.01%) and branch number (BN) ($h_{I}^{2}\overset{\wedge }{=}$ 44.33%). Epistasis of heterozygote A/G of A03_M4640 × minor-allele homozygote A/A of A08_M12338, and heterozygote A/G of A07_M11103 could significantly increase IL (Figures S2B,C). Heterozygote G/A of C09_M34850 was the major locus for increasing BN. And due to its epistasis effects, combination of heterozygote C/A of A01_M1364 × minor-allele homozygote A/A of C09_M34850 could also significantly increase BN (Figure S2D).

**Table 2:** **Predicted genetic effects of highly significant QTSs for eight traits**.

**Trait**	**Chr_SNP_Alleles**	**Effect**	**Predict**	**SE**	**−Log*****P_*EW*_***	***h*****^2^(%)**
PH	A01_M114_G/A	d	2.63	0.56	5.70	0.48
	A08_M12337_C/A	a	−3.67	0.46	14.70	1.86
	C09_M34850_G/A	a	4.59	0.47	22.20	2.92
		d	13.09	0.43	201.20	11.86
		de1	6.80	0.60	28.70	6.21
		de2	5.21	0.60	17.30	6.21
	Scaffold_M33906_G/A	a	−7.11	0.46	52.20	6.99
		d	−3.57	0.43	16.00	0.88
	A08_M12337_C/A × C09_M34850_G/A	da	−11.00	1.79	9.10	50.30
IL	A07_M11103_A/G	d	5.42	0.25	105.70	15.01
		ae2	−1.94	0.37	6.70	2.26
	A08_M12338_G/A	a	−2.76	0.26	25.00	2.60
		d	3.35	0.25	41.50	5.74
	A10_M15289_A/C	a	1.64	0.27	9.20	0.91
	A03_M4640_A/G × A08_M12338_G/A	ad	−1.98	0.33	8.70	4.02
		da	−5.50	0.96	8.00	30.92
	A07_M11103_A/G × A08_M12338_G/A	ad	−4.20	0.94	5.10	18.07
BN	A01_M1364_C/A	a	−0.20	0.04	6.10	2.08
	C09_M34850_G/A	d	0.99	0.05	101.70	25.94
		de1	0.40	0.06	9.10	4.14
SS	A08_M12212_G/A	d	1.63	0.21	14.10	6.43
		ae1	1.09	0.18	9.00	6.90
	C01_M23080_G/A	ae1	−1.54	0.18	16.90	23.13
		d	0.82	0.15	7.60	1.63
		de2	1.47	0.21	12.00	5.95
ISN	A05_M8315_A/G	a	−1.71	0.34	6.40	2.57
		d	5.06	0.57	18.40	5.62
	C04_M31883_C/A	d	8.38	0.41	89.70	15.43
	C09_M34850_G/A	d	8.12	0.40	90.10	14.48
	C04_M31883_C/A × C09_M34850_G/A	dde1	−4.54	0.60	13.20	9.04
TSW	A07_M11580_A/G	de2	0.18	0.03	8.70	4.81
	A08_M12337_C/A	a	−0.18	0.02	15.70	15.03
		de2	0.14	0.03	6.10	2.76
	C01_M30818_A/G	d	0.25	0.02	31.30	7.29
	A07_M11580_A/G × C01_M30818_A/G	dd	−0.21	0.02	18.30	10.74
BY	A07_M11103_A/G	a	−5.60	0.96	8.30	3.10
		d	8.96	0.92	21.60	1.98
		de2	23.75	1.32	71.50	27.84
	C04_M26614_A/G	d	7.79	0.95	15.60	1.50
	C09_M34850_G/A	d	7.85	0.91	17.10	1.52
SY	C04_M26614_A/G	d	2.10	0.28	13.60	2.16
	C09_M34850_G/A	d	4.26	0.27	56.90	8.94
		ae2	−2.37	0.40	8.60	5.51
		de2	4.74	0.38	35.20	5.96
	C04_M26614_A/G × C09_M34850_G/A	dde2	4.37	0.40	26.50	9.38

*Chr_SNP_Alleles, genome chromosome_SNP_major allele/minor allele; Effect, genetic effect of QTS; a, additive effect for QQ locus; −a for qq locus; d, dominance effect for Qq locus; dd, dominance epistasis; ae_2_, additive × environment interaction effect in Xiangyang; de_1_, de_2_, dominance × environment interaction effect in Wuhan, and Xiangyang, respectively; dde_2_, dominance epistasis × environment interaction effect in Xiangyang; Predict, predicted genetic effect for the QTSs; SE, standard error of predicted effect; −LogP_EW_, minus log_10_(experiment-wise P-value); h^2^, estimated heritability*.

Number of seeds per silique (SS) had very strong environment-specific additive and dominance effects ($h_{AE}^{2}\overset{\wedge }{=}$ 35.91% and $h_{DE}^{2}\overset{\wedge }{=}$ 13.66). Heterozygote G/A of A08_M12212 ($h_{d}^{2}\overset{\wedge }{=}$ 6.43%), G/A of C09_M34850 ($h_{d}^{2}\overset{\wedge }{=}$ 1.63%, $h_{de_{2}}^{2}\overset{\wedge }{=}$ 5.95%) could increase SS in different environments.

Effective silique number on main inflorescence (ISN) had high heritability ($h_{T}^{2}\overset{\wedge }{=}$ 72.57%) mostly due to dominance effects ($h_{D}^{2}\overset{\wedge }{=}$ 35.52%) of three loci (A/G of A05_M8315, C/A of C04_M31883, and G/A of C09_M34850). Based on their large main effects, these three loci could be used in selection for increasing ISN, despite the negative dominance epistasis effect between heterozygotes of C04_M31883 and C09_M34850 (Figure S2E).

Thousand seed weight (TSW) had relatively large additive and epistasis variances ($h_{A}^{2}\overset{\wedge }{=}$ 17.23%, $h_{I+IE}^{2}\overset{\wedge }{=}$ 26.07%). Increasing TSW could be expected by homozygote minor-alleles A/A of A08_M12337 ($h_{a}^{2}\overset{\wedge }{=}$ 15.03%), and also by heterozygote C/A of C01_M30818 ($h_{d}^{2}\overset{\wedge }{=}$ 7.29%), but the heterozygote of both loci should be avoided due to negative epistasis effects ($dd\overset{\wedge }{=}$ −0.212) (Figure S2F).

For biomass yield per plant (BY), the largest contributions of genetic variance were environment-specific dominance and epistasis ($h_{DE}^{2}\overset{\wedge }{=}$ 28.88%, $h_{IE}^{2}\overset{\wedge }{=}$ 27.88%). Heterozygote A/G of A07_M11103 ($de_{2}\overset{\wedge }{=}$ 23.745, $h_{de_{2}}^{2}\overset{\wedge }{=}$ 27.84%) could significantly increase BY (in environment Xiangyang). For the most important yield trait (seed yield per plant, SY), dominance and environment-specific epistasis ($h_{D}^{2}\overset{\wedge }{=}$ 11.11%, $h_{IE}^{2}\overset{\wedge }{=}$ 48.18%) were the major genetic recourses for increasing yield (Figure S2G). Heterozygote G/A of C09_M34850 could increase SY across environments ($h_{d}^{2}\overset{\wedge }{=}$ 8.94), and add extra selection response ($de_{2}\overset{\wedge }{=}$ 4.743) in environment Xiangyang ($h_{de_{2}}^{2}\overset{\wedge }{=}$ 5.96%). Although dominance of heterozygote A/G of C04_M26614 could only slightly increase SY ($d\overset{\wedge }{=}$ 2.096, $h_{d}^{2}\overset{\wedge }{=}$ 2.16%), its epistasis interaction with dominance of another heterozygote G/A of locus C09_M34850 could also have large increase for SY in environment Xiangyang ($dde_{2}\overset{\wedge }{=}$ 4.365) (Figure S2H).

### Predicted genetic effects for different genotypes

The Genotype-Phenotype (G-P) maps of epistasis SNPs for each trait, in both environments were presented in Figure S2. G-P maps exhibited various patterns as effects differed among different traits. High concordance was observed between phenotypic G-P maps and genotypic G-P maps. Based on predicted genetic effects of QTSs for each trait, we further predicted the maximum and minimum genotypic effects of the superior lines and superior hybrids in two environments on eight traits. We also predicted the genotypic effects of homozygotes (*QQ, qq*), and heterozygote (*Qq*) for eight traits in two environments, respectively (Table 3). All the predicted genotypic effects of eight traits were negative for major-allele homozygote *QQ*, but positive for minor-allele homozygote *qq*. The predicted genotypic effects of heterozygote *Qq* were positive for all the eight traits studied. Among the eight traits for all-locus heterozygote (*Qq*), the predicted genotypic values were much larger than minor-allele homozygote (*qq*) for eight traits but not for TSW in one environment. It was implied that for this rapeseed population, hybrid breeding could potentially increase breeding values of seven yield traits but not for shortening plant height.

**Table 3:** **Predicted genetic effects in two environments for genotype of ***QQ, qq, Qq***, best and superior lines, best and superior hybrids of eight traits**.

**Trait**	**Environment**	**Mean**	**QQ**	**qq**	**Qq**	**Best Line**	**Superior Line**	**Best Hybrid**	**Superior Hybrid**
PH	G+GE1	133.7	−7.55	7.55	21.63	−7.55	−16.74	4.81	−16.74
	G+GE2	134.69	−5.06	5.06	15.6	−5.06	−19.22	2.32	−19.22
IL	G+GE1	52.02	−0.58	3.83	7.66	3.83	7.11	13.68	15.32
	G+GE2	50.85	−3.69	3.69	10.06	7.94	9.51	13.68	16.59
BN	G+GE1	4.77	−0.38	0.38	1.38	0.38	0.38	1.58	1.58
	G+GE2	7.92	−0.38	0.38	0.99	0.38	0.38	1.19	1.19
SS	G+GE1	23.44	−0.45	0.45	2.59	2.63	2.63	4.72	4.72
	G+GE2	22.66	−1.26	1.26	3.33	1.26	1.26	3.92	3.92
ISN	G+GE1	57.72	−1.69	1.69	15.27	9.64	4.15	15.27	15.27
	G+GE2	50.5	−5.24	5.24	19.81	7.88	5.24	19.81	19.81
TSW	G+GE1	3.69	−0.25	0.12	0.03	0.37	0.37	0.34	0.37
	G+GE2	3.38	−0.39	0.25	0.49	0.25	0.25	0.53	0.53
BY	G+GE1	51.47	−6.29	12.91	21.73	12.91	12.91	29.56	29.56
	G+GE2	92.9	−11.79	18.41	54.18	23.2	18.41	55.83	55.83
SY	G+GE1	10.79	−1.14	1.14	6.58	1.14	1.14	8.81	8.81
	G+GE2	23.79	−3.5	3.5	16.55	3.5	3.5	16.6	16.6

*Mean, estimated mean of environment; E_1_, 2013 in Wuhan; and E_2_, 2013 in Xiangyang; QQ, homozygote of all loci with major-alleles; qq, homozygote of all loci with minor-alleles; Qq, heterozygote of all loci with Qq; Best line, predicted genotypic effect of line in the mapping population with lowest values for PH and highest values for other seven traits; Superior line, predicted genotypic effect of line in the selecting population with lowest values for PH and highest values for other seven traits; Best hybrid, predicted genotypic effect of hybrid in the mapping population with lowest values for PH and highest values for other seven traits; Superior hybrid, predicted genotypic effect of hybrid in the selecting population with lowest values for PH and highest values for other seven traits*.

There was no difference between the best lines of mapping population and the predicted superior lines for four traits (BN, SS, TSW, and SY). It was suggested that pure-line variety breeding might have only limited potential for improving these traits based on the QTSs detected for this rapeseed population. For trait IL, breeding value of the best lines was smaller than the predicted superior lines, which was due to one locus in the best line L155 (minor-allele homozygote C/C of A01_M15289). The superior line could be obtained by replacing minor-alleles to major-alleles (A/A) of A01_M15289 for increasing $a\overset{\wedge }{=}$ 1.63 in two environments and extra $ae_{2}\overset{\wedge }{=}$ 1.27 in *E*_2_. For another trait ISN, the breeding value of the best line ($G+GE\overset{\wedge }{=}$ 9.64 in *E*_1_, and 7.88 in *E*_2_) was larger than the superior line ($G+GE\overset{\wedge }{=}$ 4.15 in *E*_1_, and 5.24 in *E*_2_), because the best lines (L41 in *E*_1_ and L45 in *E*_2_) still had heterozygote locus (C/A of C04_M31883).

Among the eight traits studied, there were six traits (BN, SS, ISN, TSW, BY, and SY) with no difference of breeding values between the best hybrids and the superior hybrids in at least one environment. It was indicated that no selection advantage could be expected based on this mapping population for improving these six traits of hybrids. Expected gain of hybrid breeding could be obtained for two traits based on the best hybrids of this mapping population. For trait IL, the superior hybrid genotypes could be selected as A/G of A03_M4640 in two environments, and A/G of A07_M11103 in *E*_1_ based on the best hybrids (L155 × L76 in *E*_1_, L155 × L26 in *E*_2_) and maintained homozygote A/A for other two loci (A08_M12338, A01_M15289). There could have dramatic decrease for plant height of hybrid by just selecting all detected four QTSs of PH as major-allele homozygotes (C/C of A08_M12337, G/G of A01_M114, C09_M34850, and Scaffold_M33906).

The genotype of receptor lines and donor lines was listed in Table S2. Superior hybrid for six traits (BN, SS, ISN, TSW, BY, and SY) could be obtained via hybridization of acceptor lines (L155 and L157) to certain donor lines (L1~L154). There was only limited number of donor lines (3–13) that could contribute to target traits, except for trait ISN, which could be improved by 103 donor lines. We also found L102 was a competitive donor line of high potential. L102 could simultaneously improve four traits (SS, ISN, and SY) mainly due to its ability to donor G allele to A allele of C09_M34850, whose heterozygote could largely improve trait SS, ISN and SY with its pleiotropic effects. L102 also carried A/A of A07_M11580 and C/C of A08_M12337, which enabled L102 to improve TSW via introducing heterozygote into acceptor lines.

## Discussion

There are evidences that rare variants have large impacts on common human diseases (Cirulli and Goldstein, 2010; Yang et al., 2010; Zuk et al., 2014). Experimental evidence from association and linkage populations demonstrated that the rare genetic variation at β-carotene hydroxylase 1 (*crtRB1*) was associated with β-carotene concentration in maize kernels (Yan et al., 2010). In our study, full genetic model including epistasis and environment-specific effects was firstly used to excavate the missing heritability and dissect genetic architecture of important agronomic traits in *B. napus*. The average minor-allele frequency (MAF) in the 151 cultivars of this study was 11.07% (4.67~16.67%) for the detected 17 QTSs (10 A-alleles, 6 G-alleles, and 1 C-allele), and minor alleles had impacts on various genetic effects (−***a*** of *qq*, *d* of *Qq*, ***aa*** of *qq* × *qq*, ***ad*** of *qq* × *Qq*, ± ***da*** of *Qq* × *qq*, and ***dd*** of *Qq* × *Qq*) according to the genetic model. These minor alleles (*qq*) increased breeding values for all the eight traits and made contributions to total heritability increasing by different genetic effects.

There were total 17 main additive effects loci and 14 environment-specific additive effects loci were identified, and most of them were negative (15 negative main additive effects and 8 negative environment-specific additive effects). However, the heritability of each additive locus was quite low ($h_{ā}^{2}\overset{\wedge }{=}$ 2.27%, $h_{a}^{2}\overset{\wedge }{=}$ 0.12~15.03%; $h_{\bar{ae}}^{2}\overset{\wedge }{=}$ 5.03%, $h_{ae}^{2}\overset{\wedge }{=}$ 1.66~23.13%). It suggested that the contribution to phenotype due to negative alleles could not be neglected and the additive variances were not the major genetic contribution for most traits studied. While previous study indicated that the additional effects of the alleles originated from both parents were detected to be important for yield components traits in rapeseed (Wang and Guan, 2010). The inconsistent of results reiterated the complex genetic mechanism of yield and yield-related traits, especially for allopolyploid species such as *B. napus*. All the dominance variants were also contributed due to minor-allele heterozygotes (*Qq*). Dominance had much larger impacts on eight traits studied due to 21 main dominance loci with 19 having positive effects ($h_{\bar{d}}^{2}\overset{\wedge }{=}$ 6.14%, $h_{d}^{2}\overset{\wedge }{=}$ 0.11~25.94%) and 20 environment-specific dominance loci with 13 having positive effects ($h_{\bar{de}}^{2}$ $\hat{=}$ 4.76%, $h_{de}^{2}\overset{\wedge }{=}$ 0.30~27.84%). Which indicated that positive dominance effects were conducive to yield-related traits plasticity in *B. napus*. To data, epistatic effects were considered as important for complex traits in crops, such as plant height (Cao et al., 2001), yield (Huang et al., 2014), and salt tolerance (Wang et al., 2012) in rice seedlings, plant height in cultivated wheat (Zhang et al., 2008) and seed protein concentration in soybean (Qi et al., 2016). There were 10 pairs of loci identified with main dominance-related epistasis (*dd, ad*, and *da*) ($h_{ī}^{2}\overset{\wedge }{=}$ 19.05%, $h_{i}^{2}\overset{\wedge }{=}$ 1.35~50.30%) and eight pairs of loci detected with environment-specific dominance-related epistasis (*dde, ade*, and *dae*) ($h_{iē}^{2}\overset{\wedge }{=}$ 14.11%, $h_{ie}^{2}\overset{\wedge }{=}$ 0.74~38.80%). The most contribution of heritability for yield traits was due to 12 pairs of loci identified with epistasis between dominance effects and additive effects (*ad* and *da, ade*, and *dae*; $h_{AD+DA+ADE+DAE}^{2}\overset{\wedge }{=}$ 10.96~53.01%). It was revealed the major role of epistasis influencing rapeseed yield. None of the detected QTSs was common with the SNPs detected in the previous association studies (Li F. et al., 2014; Li et al., 2016; Liu J. et al., 2016; Liu S. et al., 2016; Wang et al., 2016; Xu et al., 2016). It confirmed again that yield-related traits are complex polygenic phenomenon in rapeseed. Four pleiotropic QTSs were found to be associated with more than one trait (Table 2). It indicated that these traits might share part of genetic basis. Thus, the pleiotropic loci should be a priority for further research, and multi-traits should be taken into account together in genetic breeding practice.

Fully characterizing the genetic mechanism mediating heterosis is helpful for increasing crop yield. While none of the current genetic models can completely explain the heterosis phenomenon. Previous study indicated that epistasis together with all levels of dominance from partial to overdominance is responsible for the expression of heterosis in rapeseed (Radoev et al., 2008). In the study, large and positive heteroses for eight yield traits were mostly due to minor-alleles in heterozygotes (*Qq*), and minor-allele homozygotes epistasis (*qq* × *Qq* of AD, *Qq* × *qq* of DA) was contributed for seven yield traits. It was concluded that epistasis together with heterozygotes loci play an important role in yield heterosis in *B. napus*.

Selection of best genotype combination is difficult due to the complexity of the genetic architecture. For example, to increase ISN, C/A of C04_M31883 × G/A of C09_M34850 should be avoided due to their negative additive effects, but their large positive dominant effects could overturn the epistasis effects and make heterozygotes the optimal choice for these two loci. Genotype-Phenotype maps of epistasis SNPs based on prediction were adopted to visually demonstrate the accumulated genetic effects of the epistasis SNP pairs. Due to the high heritability of epistasis effects, the G-P maps based on population mean exhibited a similar pattern with corresponding G-P maps based on genetic prediction. But G-P maps based on population mean may be biased away from prediction due to confounding with effects of other loci and residual error. So G-P maps based on genetic prediction could be a better choice for selection by visualizing the true effects of epistasis effects. For loci involved in multiple epistases, selection needs more caution. For IL, A08_M12338 interacted with both A03_M4640 and A07_M11103. Although G/G of A07_M11103 combined with G/A of A08_M12338 could increase IL by $ad\overset{\wedge }{=}$ 4.204, A/A of A08_M12338 should be chosen to obtain higher IL based on the total effects of the three loci. In this case, prediction function of superior line and superior hybrid is conductive to select the optimal genotype combination, thus efficiently utilize the heterosis in *B. napus*.

In the study, there were six traits (BN, SS, ISN, BY, TSW, and SY) having no difference of predicted breeding values between the best hybrids and the superior hybrids in at least one environment (Table 3). It was suggested that these six traits were already under strong breeding selection and conserved positive effects of minor-allele homozygotes (*qq*) and heterozygotes (*Qq*) for additive, dominance and epistasis effects. All superior hybrids for these six traits could be obtained via introducing heterozygote into L155 or L157, suggesting that L155 and L157 already offered suitable genetic background. Only limited amount (3~13) of donor lines could be crossed to acceptor line to gain desirable properties, except for trait ISN. It is indicated that donor lines should be chosen with caution, and that marker-assisted selection could help to reduce blindness in hybrid breeding based on the predicted breeding values.

## Author contributions

CM conceived and designed the experiments. XL, YY, and CM performed the experiments. JZ, YD and XL analyzed the data. CM and JZ contributed materials/analysis tools. JZ, CM, LZ and XL wrote the paper. JS polished the paper.

### Conflict of interest statement

The authors declare that the research was conducted in the absence of any commercial or financial relationships that could be construed as a potential conflict of interest.
