# Supplementary material for: Genomic Prediction of Genotypic Effects with Epistasis and Environment Interactions for Yield-Related Traits of Rapeseed (Brassica napus L.)
Source: Front Genet. 2017 Feb 21;8:15. doi: 10.3389/fgene.2017.00015 (PMC5318398; doi:10.3389/fgene.2017.00015)
Supplement: Supplementary file 1 [file DataSheet1.pdf]

Table S1 Predicted genetic effects and heritability of detected QTSs for eight traits.

Trait	Chr_SNP_Alleles	Effect	Predict	SE	-Log$_P$	h^2%
PH	A01_M114_G/A	a	-1.369	0.382	3.5	0.26
		d	2.634	0.556	5.7	0.48
	A08_M12337_C/A	a	-3.667	0.462	14.7	1.86
		d	1.247	0.430	2.4	0.11
		de1	1.429	0.596	1.8	0.30
		de2	-1.282	0.596	1.5	0.30
	C09_M34850_G/A	a	4.594	0.466	22.2	2.92
		d	13.089	0.427	201.2	11.86
		ae2	2.487	0.652	3.9	2.57
		de1	6.798	0.603	28.7	6.21
		de2	5.214	0.603	17.3	6.21
	Scaffold_M33906_G/A	a	-7.107	0.463	52.2	6.99
		d	-3.565	0.429	16.0	0.88
		de2	-1.738	0.601	2.4	0.31
	A08_M12337_C/A × C09_M34850_G/A	da	-11.003	1.791	9.1	50.30
IL	A03_M4640_A/G	a	-0.782	0.211	3.7	0.21
	A07_M11103_A/G	a	-1.114	0.263	4.6	0.42
		d	5.418	0.246	105.7	15.01
		ae1	0.816	0.365	1.6	2.26

		ae2	-1.939	0.372	6.7	2.26
	A08_M12338_G/A	a	-2.764	0.263	25.0	2.60
		d	3.352	0.246	41.5	5.74
		a	1.637	0.265	9.2	0.91
		ae2	1.273	0.367	3.3	1.66
	A03_M4640_A/G ×	ad	-1.984	0.330	8.7	4.02
	A08_M12338_G/A	da	-5.500	0.961	8.0	30.92
		aae1	1.627	0.374	4.9	5.41
	A07_M11103_A/G ×	ad	-4.204	0.938	5.1	18.07
	A08_M12338_G/A	dde1	-1.110	0.357	2.7	0.74
		dde2	1.286	0.356	3.5	0.74
BN	A01_M1364_C/A	a	-0.198	0.040	6.1	2.08
	C09_M34850_G/A	a	-0.184	0.049	3.8	1.79
		d	0.989	0.046	101.7	25.94
		de1	0.395	0.064	9.1	4.14
	A01_M1364_C/A ×	da	-0.915	0.221	4.5	44.33
	C09_M34850_G/A					
SS	A08_M12212_G/A	d	1.626	0.209	14.1	6.43
		ae1	1.087	0.178	9.0	6.90
		ae2	-0.485	0.177	2.2	6.90
		de1	1.259	0.292	4.8	7.71
	C01_M23080_G/A	d	-0.588	0.208	2.3	0.84

		ae1	-1.542	0.180	16.9	23.13
	C09_M34850_G/A	d	0.819	0.147	7.6	1.63
		ae2	-0.778	0.217	3.5	5.89
		de1	-0.527	0.207	2.0	5.95
		de2	1.472	0.207	12.0	5.95
ISN	A05_M8315_A/G	a	-1.711	0.338	6.4	2.57
		d	5.059	0.566	18.4	5.62
	C04_M31883_C/A	d	8.382	0.414	89.7	15.43
		ae1	1.232	0.574	1.5	3.03
		ae2	-2.321	0.568	4.4	3.03
	C09_M34850_G/A	a	-1.209	0.423	2.4	1.28
		d	8.119	0.400	90.1	14.48
	C04_M31883_C/A ×	ad	-2.249	1.021	1.6	4.44
	C09_M34850_G/A	dd	-1.750	0.426	4.4	1.35
		dae1	4.177	2.059	1.4	15.32
		dde1	-4.538	0.604	13.2	9.04
TSW	A07_M11580_A/G	a	-0.068	0.020	3.1	2.19
		d	0.058	0.022	2.1	0.40
		de1	-0.081	0.031	2.0	4.81
		de2	0.183	0.031	8.7	4.81
	A08_M12337_C/A	a	-0.177	0.022	15.7	15.03
		d	0.073	0.020	3.4	0.64

		de1	-0.059	0.029	1.4	2.76
		de2	0.140	0.029	6.1	2.76
	C01_M30818_A/G	d	0.247	0.021	31.3	7.29
		ae1	0.058	0.029	1.3	2.15
		ae2	-0.075	0.029	2.0	2.15
	A07_M11580_A/G ×	aa	-0.068	0.022	2.6	4.38
	C01_M30818_A/G	dd	-0.212	0.024	18.3	10.74
		ade1	0.151	0.061	1.9	10.96
BY	A07_M11103_A/G	a	-5.600	0.957	8.3	3.10
		d	8.959	0.921	21.6	1.98
		de2	23.745	1.316	71.5	27.84
	C04_M26614_A/G	d	7.788	0.949	15.6	1.50
		de1	-2.865	1.316	1.5	1.04
		de2	5.841	1.333	4.9	1.04
	C09_M34850_G/A	a	-4.000	0.966	4.5	1.58
		d	7.851	0.913	17.1	1.52
		ae2	-5.500	1.351	4.3	2.99
	A07_M11103_A/G ×	aa	3.309	0.985	3.1	2.16
	C09_M34850_G/A	ad	-11.183	3.283	3.2	12.35
		ade2	-16.805	4.657	3.5	27.88
SY	A04_M6147_G/A	a	-1.136	0.273	4.5	1.27
		de1	-1.088	0.369	2.5	0.58

	de2	1.086	0.376	2.4	0.58
C04_M26614_A/G	d	2.096	0.275	13.6	2.16
C09_M34850_G/A	d	4.262	0.265	56.9	8.94
	ae2	-2.365	0.397	8.6	5.51
	de1	1.313	0.372	3.4	5.96
	de2	4.743	0.378	35.2	5.96
C04_M26614_A/G ×	da	-3.765	1.086	3.3	13.96
C09_M34850_G/A	dae2	-6.276	1.525	4.4	38.80
	dde2	4.365	0.403	26.5	9.38

Table S2 Genotype of acceptor lines and donor lines for superior hybrids.

BN	A01_M1364_C/A	C09_M34850_G/A	
Superior Hybrid	A/A	G/A	
Aceptor Line L157	A/A	A/A	
13 Donor Lines[a]	A/A	G/G	
SS	A08_M12212_G/A	C01_M23080_G/A	C09_M34850_G/A
Superior Hybrid (E1)	G/A	A/A	G/A
Aceptor Line L155	A/A	A/A	A/A
Three Donor Lines[b]	G/G	A/A	G/A
Superior Hybrid (E2)	G/A	G/G or A/A	G/A
Aceptor Line L155	A/A	A/A	A/A
Three Donor Lines[b]	G/G	A/A	G/A

Aceptor Line L157	G/G	G/G	A/A
Three Donor Lines[c]	A/A	G/G	G/G
ISN	A05_M8315_A/G	C04_M31883_C/A	C09_M34850_G/A
Superior Hybrid	A/G	C/A	G/A
Aceptor Line L155	G/G	A/A	A/A
70 Donor Lines[d]	A/A	C/C	G/G
Aceptor Line L157	A/A	A/A	A/A
Four Donor Lines[e]	G/G	C/C	G/G
TSW	A07_M11580_A/G	A08_M12337_C/A	C01_M30818_A/G
Superior Hybrid (E1)	A/G	C/A	G/G
L155<br>L157	G/G	A/A	G/G
13 Donor Lines[f]	A/A	C/C	G/G
BY	A07_M11103_A/G	C04_M26614_A/G	C09_M34850_G/A
Superior Hybrid (E1)	G/G	A/G	G/A
L155	G/G	G/G	A/A
L157			
10 Donor Lines[g]	G/G	A/A	G/G
Superior Hybrid (E2)	A/G	A/G	A/A
L155<br>L157	G/G	G/G	A/A
Seven Donor Lines[h]	A/A	A/A	A/A

SY	A04_M6147_G/A	C04_M26614_A/G	C09_M34850_G/A
Superior Hybrid	A/A	A/G	G/A
L155<br>L157	A/A	G/G	A/A
Seven Donor Lines[i]	A/A	A/A	G/G

Superior Hybrids for five traits BN, SS, ISN, BY, SY could be directly obtained via hybridization of existing material. The genotype of receptor lines (L155 and L157) and its corresponding donor lines (L1~L154) were listed in this table.

[a]: L9, L22, L27, L28, L48, L55, L64, L93, L107, L118, L125, L130 or L152;

[b]: L1, L102 or L130;

[c]: L36, L40 or L137;

[d]: L1, L3, L4, L5, L7, L12, L15, L23, L24, L27, L28, L30, L32, L33, L34, L46, L47, L48, L49, L50, L51, L52, L53, L55, L58, L60, L61, L62, L64, L65, L68, L70, L74, L75, L79, L82, L84, L90, L92, L93, L95, L100, L102, L103, L108, L110, L111, L112, L113, L115, L120, L124, L125, L127, L130, L132, L133, L134, L135, L136, L137, L138, L139, L141, L142, L146, L149, L151, L152 or L154;

[e]: L2, L21, L42 or L143;

[f]: L1, L11, L16, L26, L28, L37, L41, L42, L69, L89, L99 L102, L125

[g]: L12, L14, L15, L46, L63, L71, L92, L103, L148, L153;

[h]: L10, L116, L121, L128, L140, L145, L147;

[i]: L36, L43, L47, L77, L91, L102, L115.

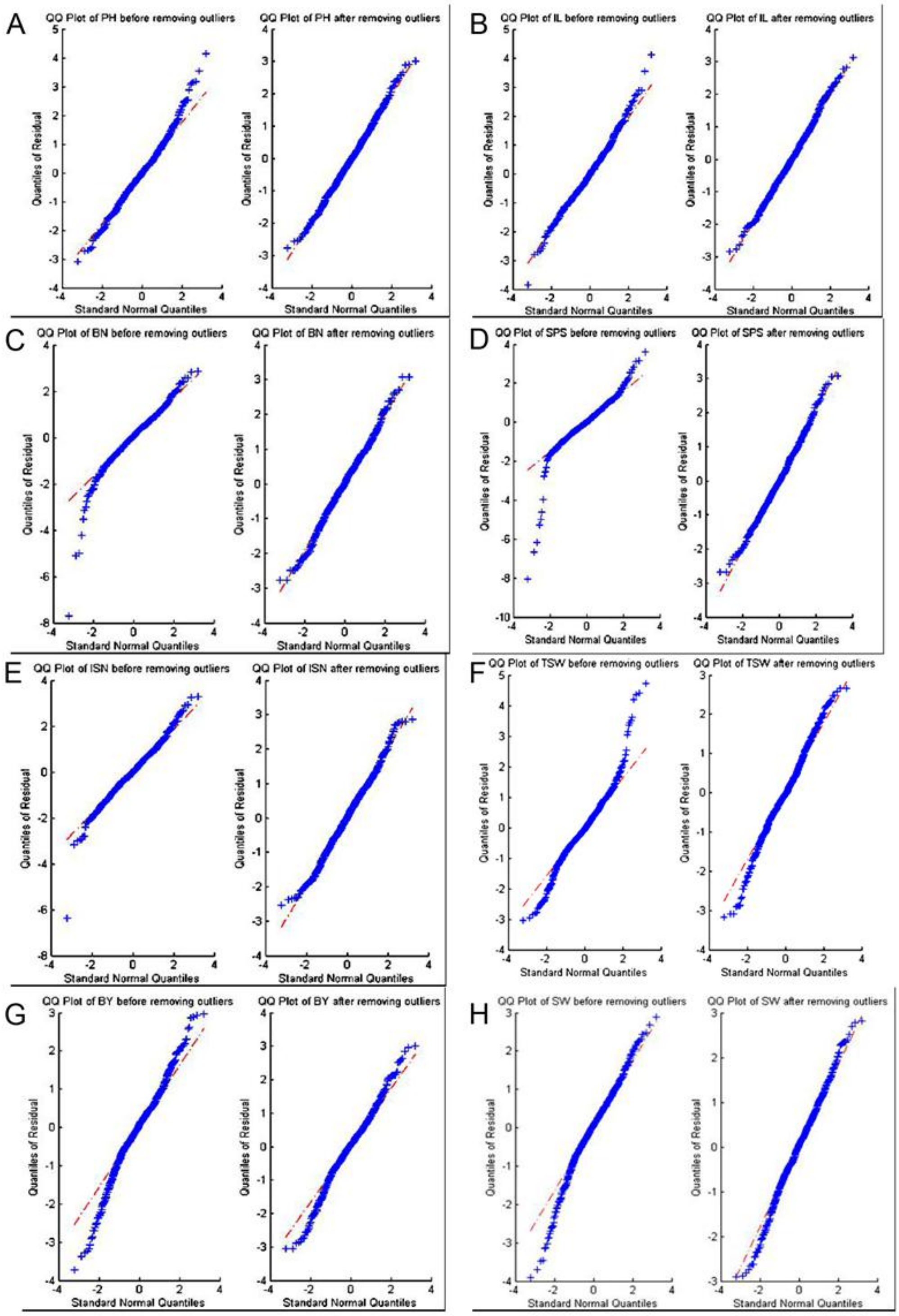

Figure. S1 QQ plot of predicted residual effects for each trait before and after removing outliers.

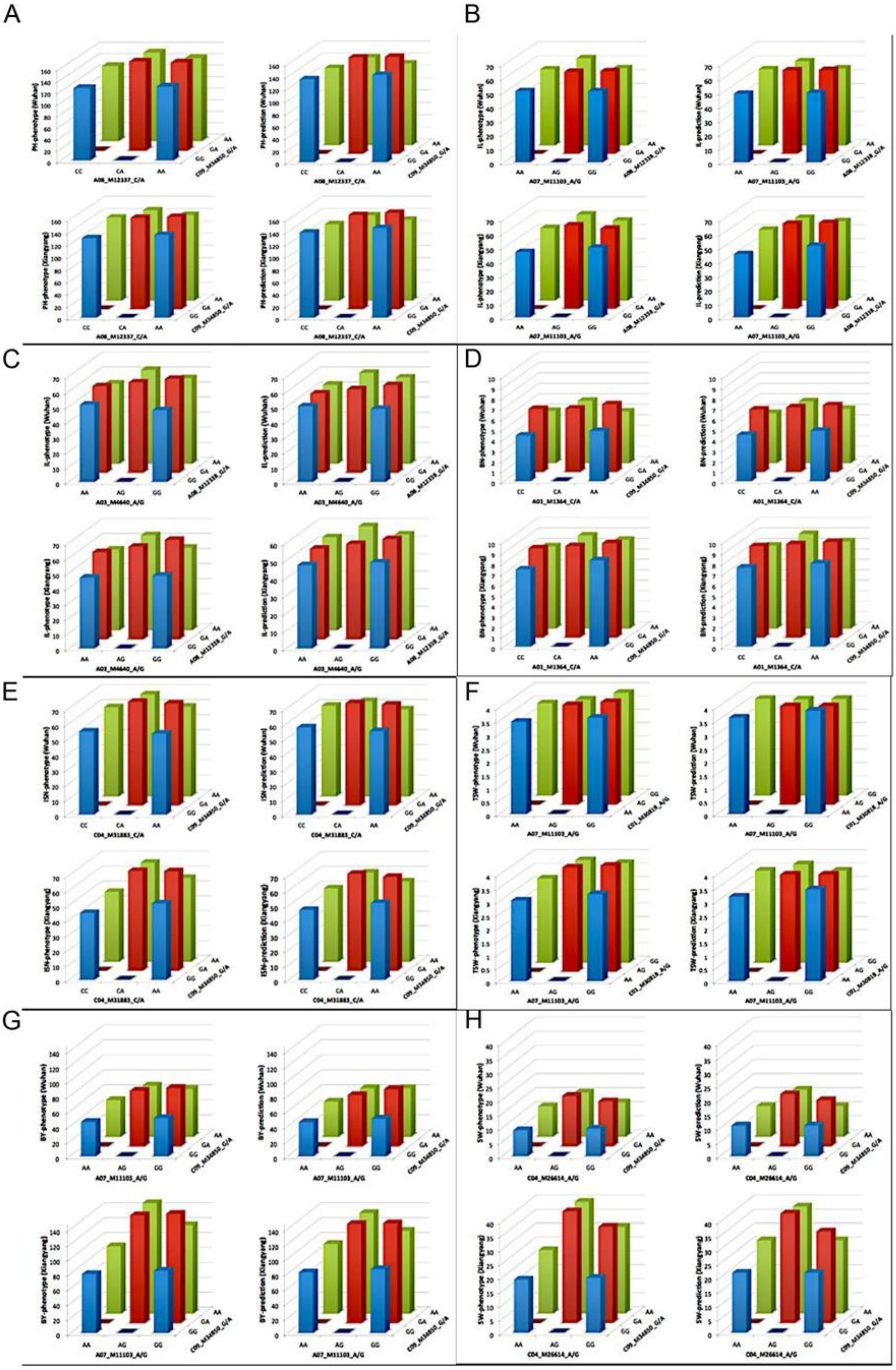

Figure. S2 Genotype-Phenotype map of epistasis SNPs for each trait. In the left panels, each

bar represents the population mean in that genotype class for corresponding trait. In the right panel, each bar represents the sum of all predicted effects (μ, main effects and epistasis effects) in that genotype class for corresponding trait.
